# What Matters Most to People Living With Dementia and Their Care Partners During Emergency Department Visits

**DOI:** 10.1111/jgs.70238

**Published:** 2025-12-08

**Authors:** Clark Benson, Kayla Dillon, Laura Block, Kristin Merss, Valentina Flores Diaz, Susie Fernandez de Cordova, Maria Mora Pinzon, Cameron Gettel, Manish N. Shah, Andrea Gilmore‐Bykovskyi

**Affiliations:** ^1^ BerbeeWalsh Department of Emergency Medicine University of Wisconsin‐Madison School of Medicine & Public Health Madison Wisconsin USA; ^2^ School of Nursing, University of Wisconsin‐Madison Madison Wisconsin USA; ^3^ Department of Medicine, Division of Geriatrics and Gerontology University of Wisconsin‐Madison School of Medicine & Public Health Madison Wisconsin USA; ^4^ Department of Emergency Medicine, Yale School of Medicine Yale University New Haven Connecticut USA

**Keywords:** dementia, emergency department, outcome measure

## Abstract

**Introduction:**

Of the 6 million people living with dementia (PLWD) in the United States, half visit an emergency department (ED) annually. Little is known about the specific emergency care preferences and priorities of PLWD and their care partners. This descriptive qualitative study engaged PLWD and care partners to identify their ED care priorities and the factors that influence their overall evaluation of ED care.

**Methods:**

We recruited PLWD receiving care in a large academic ED and their care partners to participate in individual or dyadic interviews. Interviews were analyzed using thematic analysis and member checking interviews were completed to confirm and expand on study findings.

**Results:**

We conducted interviews with 55 participants (*N* = 19 PLWD, 24 care partners, 6 dyads). PLWD and care partners evaluated ED care experiences through a summative lens shaped by: (1) universal priorities common across all participants and (2) individual values that varied in importance and quality between individuals. Universal priorities included feeling respected, clear communication, and being informed about and involved in their emergency care decisions. Individual values included preferences around the who and how of decision‐making, attention to cognitive health, and degree of escalation of care. Several contextual factors shaped the appraisal of ED visits including the timing of evaluation and nature of the precipitating event (acute/unknown cause vs. chronic/known or suspected cause).

**Conclusion:**

Findings suggest that interpersonal interactions, including being informed about care and involved in decisions, strongly influence the evaluation of ED visits for PLWD. These findings can support the development of person‐centered outcome measures capable of evaluating these priorities.

## Summary

- Key points
  ○ Emergency department patients living with dementia and their care partners identified universal priorities including knowing who is providing their emergency care, receiving respectful and compassionate treatment, and being informed in a manner attuned to cognitive changes.
  ○ Individual values such as preferences for involvement in decision‐making, care intensity, and cultural‐linguistic congruence are more variable and shape participants' evaluations of their emergency care experience.
  ○ While both universal and individual priorities shape emergency care experiences, the interpersonal quality of care–particularly whether participants felt respected—was a dominant driver for overall satisfaction, even when other care priorities were not met, suggesting that more sensitive outcome measures of emergency care for populations with dementia should incorporate a breadth of domains.
- Why does this paper matter?
  ○ This study directly engaged people living with dementia and their care partners to identify what matters most to them during their emergency care experience. Findings highlight the importance of including people living with dementia in research aimed at improving emergency care quality and offer a vital first step for developing dementia‐sensitive outcome measures to evaluate and improve emergency care quality.

## 1 Introduction

Over 6 million people in the United States live with dementia, and more than half visit an emergency department (ED) each year [1]. This frequent ED use reflects, in part, inadequate support for managing dementia‐related symptoms and coexisting health conditions. The ED provides critical health and social care access for people living with dementia (PLWD) experiencing acute or chronic medical exacerbations or distressing behavioral and psychological symptoms [2].

Given the importance of ED care for PLWD, there is a critical need to ensure that ED care is not only adapted to the needs of the population, but that dementia‐specific ED care can also be sensitively evaluated. Current research indicates hospitalization and re‐hospitalization, mortality, falls, delirium, and care partner strain are associated with PLWD visiting the ED [1, 3]. These outcomes are often influenced by underlying frailty or advanced illness and may not reflect care improvements. Use of the ED may signal disease progression or palliative needs, making conventional outcomes like re‐visits or mortality less relevant to what PLWD value [4, 5].

The 4Ms Framework of an Age‐Friendly Health System [6, 7] emphasizes identifying “What Matters” to older adults in considering and responding to their health care goals. Yet little is known about the priorities of PLWD during ED visits, limiting our capacity to systematically evaluate what matters most for emergency care in practice and research [4, 8, 9]. This gap is especially pressing for minoritized racial and ethnic groups, who are disproportionately affected by dementia and underrepresented in research [10] and have unique constraints in healthcare access, leading to disparate ED visits [11]. Language barriers further complicate communication, reducing the likelihood that ED staff engage care partners in decisions [11].

Through interviews with English‐ and Spanish‐speaking ED patients living with dementia and their care partners, this study aims to explore what matters most to PLWD during an ED visit to inform the development of a person‐centered outcome measure. The intended purpose of this measure is to support the evaluation of ED visits and the identification of ED care practices that may require adaptation in the context of cognitive impairment.

## 2 Methods

### 2.1 Design

This descriptive qualitative study employed semi‐structured interviews for data collection and analytic techniques from reflexive thematic analysis. The study was approved by the University of Wisconsin‐Madison Institutional Review Board (2023‐0312).

### 2.2 Sample

We recruited PLWD and/or their care partners from a large midwestern academic ED between August 2023 and December 2024. The study team included individuals integrated with the clinical ED care environment to facilitate initial approach and recruitment of participants as well as English‐ and native Spanish‐speaking individuals trained in the evaluation of capacity to consent to research, semi‐structured interviewing techniques, and thematic analysis. We included patients with any dementia diagnosis confirmed through electronic medical record review or proxy informant (e.g., clinical staff, care partner, or family member) that had an ED visit, spoke English or Spanish, and had capacity to understand the voluntary nature of the study and share their perspective on their ED visit. Capacity was confirmed via the Evaluation to Sign Consent questionnaire and ongoing assessments of PLWD comfortability and recollection of their ED visit [12]. Any current or former family care partner to a PLWD who had an ED visit that spoke English or Spanish was eligible. For dyadic participation, informed consent of all individuals was required. As with individual interviews, all PLWD were required to have the ability to communicate their views about their ED experience and understand the voluntary nature of the study before and while participating in a dyadic interview, assessed through the Evaluation to Sign Consent Questionnaire and ongoing assessments [12].

### 2.3 Data Collection

Participants completed either individual or dyadic interviews (Text S1). Based on their preference, interviews were conducted in the ED (10–20 min) or within 3 months post‐visit by phone or in‐person (30–60 min). An interdisciplinary team with expertise in dementia care, emergency care, and approaches to interviewing PLWD created the interview guide, broadly covering priorities and expectations of ED care with the goal of understanding what matters most for PLWD during an ED visit. Interviews were transcribed and analyzed in the language in which they were performed.

### 2.4 Data Analysis

**FIGURE 1:**
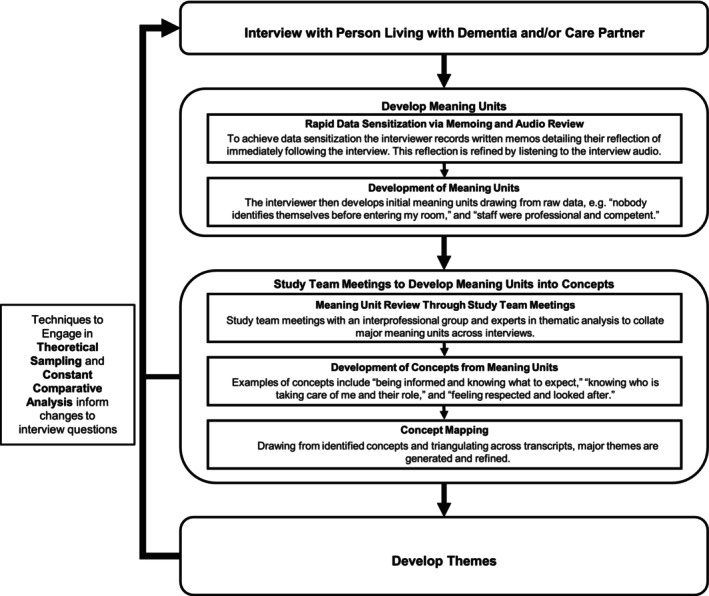
Analytical process and examples.

We used iterative reflexive thematic analysis, which emphasizes deep engagement with the data and inductive theme development [13, 14]. Our process included memoing, team meetings, concept mapping, constant comparative analysis, theoretical sampling, and member checking (Figure 1; Table 1).

**TABLE 1:** Summarized data analysis steps.

Activity	Timing	Purpose	Team members
Rapid data sensitization	Immediately after interview	Interviewer immediately writes a memo on the key meaning units from the interview, roughly transcribing the interview for later analysis	C.B., K.D., L.B., K.M., V.F.D., S.F.C. (interviewers)
Data analysis	About a week before a study team meeting	Study team independently reviews interview audio file and transcript to note key meaning units	A.G.‐B., C.B., K.D., L.B., K.M., M.M.P., V.F.D., S.F.C.
Study team meetings	Weekly for the first 15 participants Biweekly to ad hoc for later participants	Interviewer leads study team meeting to describe major meaning units from the interview, developing concepts from meaning units	A.G.‐B., C.B., K.D., L.B., K.M.
Concept mapping	Biweekly after initial concepts were established	Relationships between concepts are explored to facilitate the development of themes from concepts	A.G.‐B., C.B., K.D., L.B., K.M.
Constant comparative analysis	After several interviews had been completed and analyzed	Compare and scrutinize differences between interviews to identify latent contextual factors that impact participant experiences and perspectives	A.G.‐B., C.B., K.D., L.B., K.M.
Aspects of theoretical sampling	After several interviews had been completed and analyzed	Identify concepts and themes that are lacking in description and modifying interview questions and sampling to fill these gaps	A.G.‐B., C.B., K.D., L.B., K.M.

Data analysis began with rapid data sensitization through a combination of memoing and reviewing interview data. Immediately following an interview, interviewers completed memoing on context and salient impressions to share with the team [15]. We then analyzed interview audio and transcripts to rapidly identify major meaning units for further discussion.

At team meetings, meaning units were developed into concepts through consensus. Once several concepts were developed, we began concept mapping to relate concepts to each other and develop concepts into themes. Concept mapping sets a central concept as a diagram, drawing from memos, transcripts, and previous meeting notes to detail related concepts or variations within a concept to better capture the dimensions and complexity of data [16].

Techniques from comparative analysis and theoretical sampling facilitated the identification of contextual factors shaping participant experiences and determined new lines of questioning or sampling to pursue. While this is not a grounded theory study, these techniques allowed us to maximize our understanding of variation in the situated realities of PLWD and care partners [17, 18, 19]. Drawing from the constant comparative method, we developed case summaries of each interview to scrutinize and compare characteristics specific to each individual ED visit that may modify evaluation [20, 21]. We drew from aspects of theoretical sampling by targeting participants with experiences not represented in the data and by adjusting interview questions to probe for concepts lacking detail. For instance, one participant mentioned how arriving at the ED in an ambulance reduced their wait time and improved their ED experience. In subsequent interviews, we probed other participants to understand if the mode of arrival influences ED evaluation.

**FIGURE 2:**
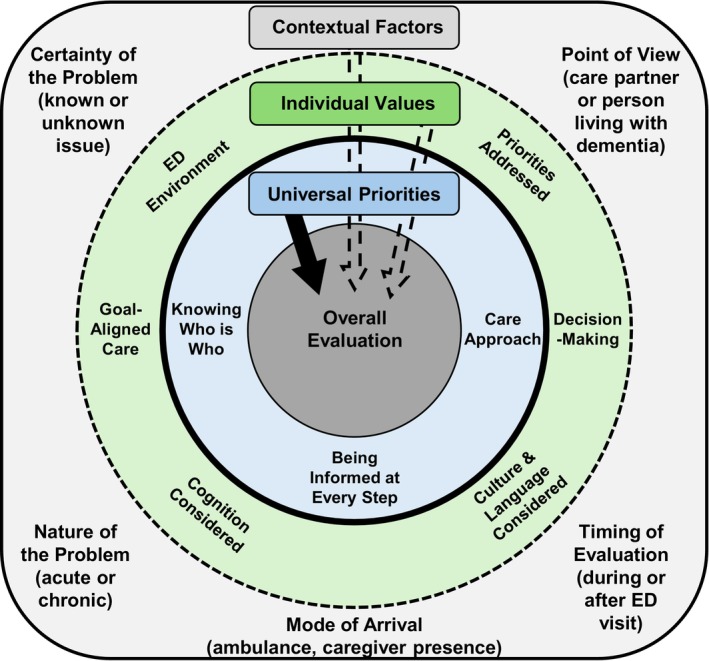
Model of how universal priorities, individual values, and contextual factors impact emergency department patients living with dementia and their care partners' overall evaluation of their emergency department visit.

To enhance trustworthiness, we used audit trails to increase dependability, reflexive journaling and peer debriefing to facilitate reflexivity, and member checking to strengthen confirmability of findings [22, 23, 24, 25]. Member checking was completed through follow‐up interviews with eight participants (*N* = 1 dyad, 4 PLWD, 2 care partners) within 3 months of their initial interviews. During these sessions, we shared preliminary interpretations of the data, including an early version of the conceptual model presented in Figure 2, and solicited participants' feedback on whether the findings reflected their ED experience (Text S2).

## 3 Results

Of the 55 participants, 51 (*N* = 23 PLWD, 28 care partners) were English‐speaking while four (*N* = 2 PLWD, 2 care partners) were Spanish‐speaking. Participant demographic characteristics are presented in Table 2. Interviews averaged 27 min in length. Most PLWD provided overall summative evaluation statements of their visit. Sixteen PLWD rated their experience positively, five were undecided, and one negatively evaluated their experience. Figure 2 represents a model outlining the factors that influenced participants' overall evaluation of ED care. A summary of findings is provided in Table 3 (see Table S1 for an expanded version and Table S2 for quotes in Spanish).

**TABLE 2:** Demographic data.

		PLWD	Care partners
Total (including dyadic interviews)		25 (45%)	30 (55%)
Age	Average (min–max)	76 (56–88)	63 (30–95)
Ethnicity	White	19 (79%)	26 (90%)
	Black or African American	0 (0%)	1 (3%)
	Hispanic	2 (4%)	2 (3%)
	American Indian	0 (0%)	1 (3%)
	Not reported	2 (8%)	0 (0%)
Gender	Female	13 (54%)	19 (66%)
	Male	11 (46%)	11 (38%)
	Other	0 (0%)	0 (0%)
	Not reported	1 (4%)	0 (0%)
Education	High school	5 (21%)	2 (7%)
	Some college	2 (8%)	2 (7%)
	Technical school	4 (17%)	4 (14%)
	4‐Year college	6 (25%)	10 (34%)
	Post college	10 (42%)	11 (38%)
Timing of interview	During ED visit	14 (58%)	14 (48%)
	After ED visit	10 (42%)	20 (69%)

**TABLE 3:** Findings by priority level, theme, and supporting quotes from participants.

Universal Priorities: What matters most. Shared and uniform	
Theme	Quotes
Being informed at every step	“And you're, you're there and you're wondering, you know, what is that like. What am I waiting for? Um. Is there good news or bad news. That sort of thing. So it's just very indefinite.” (Care Partner 3) “With some [PLWD], some significant percentage of their fears can be allayed to some extent if they're given a little bit of explanation of this is what we're trying to accomplish, this is how long it'll probably take roughly between that kind of thing. Explanation of what they're doing and you kind of know what they're doing, but a little bit more explanation would be helpful, I think.” (PLWD 4)
Knowing who is taking care of me and their role	“To know who I'm seeing, their name, and what they're affiliated with. You know what type of doctor they are. And every one of them has told me you know, which is important to me. So I would like to know that.” (PLWD 15) “Once you get into a room, people come in and out very quickly and they don't identify themselves, they don't give their name or say if they are a nurse or a P.A. or a doctor or a resident or an attending or a specialist. Nobody identifies themselves, and it's all kind of a blur.” (PLWD 10)
Care approach	“I thought the, um, staff, you know, overall were very professional and competent. And so if you don't think the people who are trying to help you are both kind and competent, it's, it's problematic. But I thought, I thought they were, you know, treated me very in a very kind and helpful fashion and were very professional and competent.” (PLWD 4) “They have always treated me with goodness, patience and I would say with mercy too. They have a lot of patience. They cover me, like that day when I arrived with chills, they put warm sheets on me and everything.” (Spanish‐Speaking Dyad 1—PLWD)
Values and Preferences: More variability. Individually specific in some instances.	
Goal‐aligned care	“The E.D. seems better, I guess maybe they seem a lot better than primary care doctors. They spend more time. That's just like kind of how they address problems, because I think they look to fix the problem, you know, not just say, well, I'm not feeling well, so I'll send her home and give her a few more days to see if she gets worse. So, you know, that's kind of where we're at with your primary doctor. I've seen it with my own family, too. They get older. The primary doctors don't want to do as much.” (Care Partner 2) “They gave her a prescription for antibiotics for a urinary infection. She had no signs of fever, discomfort, pain, anything. And so, we went home. We had to get the antibiotics from the pharmacy and start her on that. And 2 days later, I got a call from somebody in the organization, ‘Does she have any of these symptoms?’ I said ‘No.’ They said, ‘Stop taking the antibiotics because you know you can develop resistance to them.’ So, as I said, I thought all of this was kind of over the top.” (Care Partner 3)
Decision‐making	“I should be the sole one that decides about what my care is. Obviously, I can't decide the critical medical things that need to be done. I don't know. I'm not a doctor. I don't know what's going on inside my brain, inside of my body. But I just want to be included.” (PLWD 5) “You'll find the need to have a family or friend or neighbor that can answer some questions that maybe the patient stalled on.” (Dyad 1—Care Partner)
Cognitive health‐related needs taken into consideration	**Interviewer:** “And would there be anything you'd want people who care for you to know about just that you are experiencing memory changes?” **PLWD:** “They know. I try not to give them everything about me and my health, I just don't want them to worry, but they are aware that I have a memory problem and they cooperate with me on that.” (PLWD 2) “I don't know that they always know? She's not there for memory issues. She's there usually for gastrointestinal type things or pain. So I don't know if they put a note in the chart that this person has memory issues. If they do, then explaining, repeating themselves, I'm here to do this and then come back. I have to go and get something. When I come back, I'm here to do this. So that helps. But again, I don't know if they know to do that because I don't know if they know she has memory issues.” (Care Partner 23)
Culture and language considered	“Usually … I do the interpretation. If it's something that's technical … if it has something to do with the brain, let it be the scientific words that I don't know how to translate, then I let the interpreter translate it, but I then explain it to him [PLWD] anyway … That's what I do because sometimes, even though there are interpreters, well they have a way, the dialect is different, and you can tell.” (Spanish‐Speaking Dyad 2—Care Partner) “While in the ER [interpreter] was one per tablet. But since he [PLWD] didn't hear well and didn't have his hearing aids, then… I had to ask him questions, then I answered it, I listened to him or asked him the question, I simplified it or answered him then, because after that there was no longer a need for an interpreter.” (Spanish‐Speaking Dyad 2—Care Partner)
Priorities are addressed	“Today things have gone well. I mean she came in by ambulance so they got her in a room right away which is good, and then they started the bloodwork and urine tests and everything right away. So they got things moving pretty quick, got the results back and things and a kind of a game plan I guess. And it's gone really smoothly today. Quickly. I've been sitting here all day which is great [laughs].” (Care Partner 2)
Environmental preferences	“You've got three very different conversations happening at the same time. For somebody who has memory problems that can be concerning. So somehow making it more, insulated, or somehow keeping the noise down. I think because when we transitioned to the neurology area, they had a nurse right outside my mom's room and it was quiet. The lights were low. I mean, it was like definitely they were focusing on that kind of a patient. And she settled right down…” (Care Partner 8)

Across interviews, both patients and care partners emphasized the importance of being viewed as a care unit rather than as individuals in the ED. The care unit is comprised of the PLWD, family members, and care partners, and was described as an interdependent entity as the ED experience affects not only the individual with dementia, but also unpaid family or friend care partners. This perspective was especially salient among Spanish‐speaking participants, who more strongly expressed the need for care teams to include care partners and family members in decisions: “…she [PLWD's daughter] is the one who battles with me, when I am in pain” (Spanish‐Speaking Dyad 1—PLWD).

### 3.1 Universal Priorities

Most PLWD and care partners shared a set of universal priorities that played a central role in how they evaluated their ED experience. Three universal priorities were identified: (1) Being informed on results, wait times, and what to expect; (2) Knowing who is taking care of them and their role; (3) Care approach and being treated respectfully and kindly. Participants shared that when these priorities were sufficiently addressed, their overall evaluation of the ED visit tended to be positive even if some aspects of care fell short. For example, one PLWD described their experience favorably due to the compassionate care they received despite expressing dissatisfaction with the level of information provided about the rationale for certain procedures. A common thread across all universal priorities is the desire for frequent and high‐quality engagement with the PLWD, which should be considered for future measure development.

The most commonly mentioned universal priority across both PLWD and care partners was *being informed on their results, wait times, and what to expect* early, often, and consistently throughout the ED visit. One PLWD negatively evaluated their ED visit due to the lack of communication: “They were making all kinds of decisions without explaining anything to me” (PLWD 10). Several PLWD affirmed that communication was most important to them in the ED:

Interviewer When you're in the emergency room, what is most important to you?

PLWD 17 Communication. Letting me know what they're doing and what's going on and what's wrong with you.

Another universal priority was *knowing who is taking care of them and their role*. Due to the volume of staff entering and exiting the PLWD's room during their ED visit, participants appreciated when ED staff identified themselves and explained what they were going to do. One care partner noted the challenge of knowing who is who: “One thing that I find difficult is you don't know … who's in charge. People are coming in and out … but you're not sure who a physician is and what their role is” (Care Partner 3). A PLWD added the need to know the name, affiliation, and role of who is in the room and why.

Finally, participants described the *care approach* as a universal priority during an ED visit. Most participants value ED staff who were patient, respectful, competent, and kind: “The doctors, they're dedicated to their job and dedicated to their patients. You couldn't ask for a better doctors or better care than right here… They come in smiling. And I love it” (PLWD 15). Some participants mention the important calming effect a good care approach has:

This past, most recent doctor was very kind and patient and cheerful. And that was very important to her [PLWD] and her family … because it does calm a person. It calms me, it calms her … You feel respected and heard. (Dyad 1—Care Partner)

### 3.2 Individual Values

Participants identified six individual values, which did not *typically* have as much influence over the participant's overall evaluation of the ED visit compared to universal priorities yet were still important factors shaping their ED visit: (1) goal‐aligned care; (2) decision‐making; (3) cognitive health‐related needs taken into consideration; (4) culture and language considered; (5) priorities are addressed; and (6) environmental preferences.

Attention to *goal‐aligned care*, specifically the intensiveness of desired care, was important to some PLWD and care partners. Others deferred to ED staff prerogative, feeling comfortable with the ED staff performing any procedure or test they thought necessary. Specific and early communication with the PLWD and care partners on their desired level of care helped to ensure ED care was aligned with the PLWD's overarching goals of care:

The question is, how much do you have to do? If you want to be a physician who doesn't make any mistakes, you order every test in the book. The fact that she does have a DNR bracelet on, and if she came in with a wound and nothing else, why not just treat that? (Care Partner 3)

Preferences for involvement in *decision‐making* around which procedures and tests to perform, as well as next steps including hospitalization or returning home, varied across PLWD and care partners. Some PLWD wanted to remain their own primary decision‐maker, whereas others preferred involving their family. Importantly, all PLWD wanted to be involved in their own decision‐making. When care partners held healthcare power of attorney, some still involved the PLWD, while others opted for private discussions with staff. Identifying decision‐making roles early enables individualized approaches to inclusion, support, and participation in decision‐making processes.

Several PLWD and care partners mentioned that staff being aware of the PLWD's changes in memory was important to them, underscoring the importance of taking *cognitive health‐related needs into consideration*. Specifically, PLWD preferred ED staff to be aware that they might miss or forget key information and need the information to be repeated often or stated differently in a way they could understand. This was particularly relevant for individuals with moderate dementia or longstanding cognitive impairment. Awareness of these needs allowed staff to adjust communication, such as repeating information or avoiding medical jargon, to better support understanding and engagement:

I can't pronounce words anymore. I don't know the meaning of them and that's been pretty new … Show pictures to me if it's at all possible. But like I said, if they don't talk medical terms, then I can understand what they're saying. (PLWD 15)

Non‐white and/or Spanish‐speaking participants valued having their *culture and language considered* in the ED. This consideration generally revolves around ensuring ED staff can effectively communicate with non‐English‐speaking PLWD:

My mom is Japanese. So sometimes I'm worried that if people keep using big words, it's hard to communicate. You got to keep things simple and to the point. She does have an accent, and it's probably harder for other people to understand her. (Care Partner 16)

Spanish‐speaking participants specifically mentioned challenges with receiving information in Spanish, especially disliking the use of interpreters and discharge documents in English:

Yes, they have done it [interpretation] with a tablet, that is dangerous for me. I have always told them to contact me…If you don't know the person, the person's history, intimately like a family member. That you can explain to him. Knowing what the person knows, what they understand, what they like it's not the same, right? (Spanish‐Speaking Dyad 2—Care Partner)

Some PLWD and care partners felt it important that their *priorities are addressed* in a timely manner. This was particularly important for needs including pain management, hydration, and managing temperature as soon as possible. Care partners contributed to this theme more than PLWD.

Finally, *environmental preferences* varied among PLWD and care partners. Some participants mentioned the potential value of having the emergency room environment adapted to their preferences, specifically the noise and lights often present in the ED: …your emergency room is what it is. There's all kinds of stuff happening. But just for someone with memory loss, the noise and the light are real important to keep them stable” (Care Partner 8). Another participant commented on the helpfulness of visual markers: “It's important to have everything visually marked. So the person knows where they are and where they are going. A lot of that can be done with color and numbers (PLWD 11).

### 3.3 Contextual Factors Shaping Care Evaluation

We identified several contextual factors that influenced how participants evaluated their ED visit. These included the participant's level of certainty about their medical issue prior to arrival, the nature of the problem, whether the person was a PLWD or a care partner, the mode of arrival, and the timing of the interview. PLWD who frequently visited the ED for known issues tended to be more critical of their care, as they were more likely to know necessary tests and procedures and have pre‐set expectations for their encounter as a result. In contrast, PLWD presenting with acute, unfamiliar issues were typically less critical, often expressing trust in the care team with sentiments such as “they're doing the best they can.” The timing of the interview also influenced evaluations. PLWD interviewed during their ED stay, especially those who arrived via ambulance, were often more positive or uncertain in their assessments, possibly due to heightened stress. Comparatively, PLWD interviewed after their ED visit were more reflective and critical, more readily identifying aspects of their ED visit that did not go well. Finally, contextual factors sometimes aligned with priorities and values. For example, PLWD who were more certain about their issue(s) often placed greater importance on receiving care aligned with their goals, compared to those who were unsure of their issue(s).

## 4 Discussion

This study offers new insights into what matters most to PLWD during ED visits. Participants prioritized being consistently informed, knowing who was caring for them, and receiving respectful, compassionate treatment. Some values were especially important to certain participants, such as staff awareness of their dementia diagnosis and alignment of care with their personal goals, particularly when these diverged from the intensive, protocol‐driven nature of typical ED care.

Many of the priorities identified by PLWD in our study mirror those of ED patients without dementia, particularly older adults. Findings from several other studies demonstrate that valuing respectful treatment, clear communication, involvement in decision‐making, and care that aligns with their overarching goals is common across older adult ED patients with and without dementia [8, 11, 26, 27, 28, 29, 30, 31, 32]. Being kept informed throughout the ED stay, especially regarding wait times and test results, has been commonly reported as important for ED patients without dementia [33, 34, 35, 36, 37]. Confusion about the roles and responsibilities of ED staff is also prevalent among all ED patients and has been linked to communication breakdowns, feelings of isolation, and suboptimal care when patients are unsure whom to approach for assistance [38, 39]. These shared priorities are reflected in hospital quality measures for older adults, such as the CMS Age‐Friendly Hospital Initiative, which includes elicitation of healthcare goals that assess how effectively hospitals honor older patients' health‐related goals and treatment preferences [40].

While what matters most to PLWD and their care partners in the ED align well with those identified by ED patients with and without dementia in other studies, our findings identify ways specific care approaches may need to change in the context of cognitive impairment. Notably, PLWD emphasized the importance of especially frequent, consistent communication regarding their care, including clear identification of ED staff and explanations of their roles and actions every time they enter the room—ideally delivered by the same providers when feasible. Participants also stressed the need for staff to recognize and accommodate cognitive impairment by adjusting communication strategies, such as avoiding medical jargon and repeating key information to support comprehension and decision‐making. Overall, we found that ED patients living with dementia place a uniquely high level of importance on routine information provision in the ED. Attention to these priorities in future research can potentially improve the sensitivity of emergency care‐specific outcome measures for trials and interventions, which largely focus exclusively on mortality and utilization endpoints [1, 4, 41, 42].

Other studies across diverse healthcare settings have similarly identified the importance of interpersonal aspects of ED care, particularly patient involvement in decision‐making and close communication with involved care partners who are providing support and advocacy for the patient during an ED visit [11, 26, 27, 38]. These findings underscore the necessity of developing evaluation tools that both explicitly address the quality of interpersonal interactions, as well as capture other dimensions of care encounters that are meaningful and could otherwise be overlooked, such as inclusion in decision‐making. In the context of evaluating emergency care for PLWD, multidimensional evaluation will be particularly important and should ideally encompass both patient experience metrics as well as dementia‐specific domains.

Decision‐making was a key value, though preferences for who should be involved varied. All participants emphasized including the PLWD in care decisions, but some preferred broader family involvement, especially among Spanish‐speaking participants. These individuals often viewed the PLWD and care partners as a care unit and preferred culturally responsive communication. This differs from some English‐speaking participants and aligns with other findings from Spanish‐speaking PLWD care partners [11, 43]. These differences support the use of supported decision‐making frameworks in EDs, which facilitate the designation of trusted others by those with cognitive challenges to assist in care choices [44, 45].

Finally, study findings provide a rich array of potential measurement domains to inform tools that address the need for a within‐ED evaluation beyond clinical outcomes [9, 11]. Given the variability in dementia severity and ED experiences, evaluation tools should be flexible—allowing respondents to rate the importance of specific goals, whether those goals were met, and incorporating contextual factors like timing, setting, and reason for the ED visit. Consistent with prior research, PLWD assessments during and shortly after visits were generally aligned [46]. Likely due to reduced stress and greater clarity, evaluations completed after the ED visit tended to be more critical, while evaluations completed during and shortly after the ED visit were more often uncertain in their evaluation and described more specific nuances of the ED, such as challenges navigating the ED hallways. We also observed differences between PLWD and care partner perspectives, with care partners offering more critical evaluations. This underscores the importance of obtaining input directly from PLWD and calls for further research systematically comparing the ED experiences and priorities of PLWD and care partners, as well as exploring variation in responses among PLWD at various stages of disease and with varying levels of functional communication. Prior studies demonstrated PLWD in mild‐to‐moderate stages provide reliable answers about their feelings, experiences, and preferences [47, 48]. Two studies specifically found that people living with mild‐to‐moderate dementia could consistently and accurately provide responses to fact‐based items related to their experiences and quality of life over a two‐week period [47, 48]. The salience and impact of an ED visit, and whether views may change upon conclusion of the visit is less well understood and merits further investigation.

## 5 Limitations

This study was conducted at a single, highly ranked academic ED with faculty specializing in dementia care, which may have contributed to more favorable participant evaluations of overall ED care. While this dementia‐specific awareness may have reduced potential biases among staff towards patients with dementia, important variability exists and staff perspectives towards PLWD and care partners and the influence on communication patterns, engagement, and decision‐making should be explored in further studies [48, 49, 50]. The site also serves a predominantly white, educated, English‐speaking population. As a result, recruitment required extended efforts and ongoing adjustments to better include culturally and linguistically diverse participants, particularly those with lower rates of formal dementia diagnoses. The inclusion of Spanish‐speaking participants highlighted differences in family involvement and underscored the need for culturally responsive care. Future research should explore how priorities differ across minoritized groups, whose unique experiences are critical to understanding and improving ED care for all PLWD.

## 6 Conclusion

Despite varied experiences, PLWD and their care partners expressed consistent priorities for ED care. These priorities include being well‐informed, being treated with kindness and respect, and knowing who is providing their care. Our findings suggest a foundation for developing more sensitive outcome measures that capture the full scope of what matters to PLWD, helping identify meaningful improvements in geriatric emergency care.

## Author Contributions

A.G.‐B., C.B., L.B., and K.M. contributed to the study concept and design; C.B., K.D., L.B., K.M., V.F.D., and S.F.C. contributed to the acquisition of subjects and/or data; A.G.‐B., C.B., K.D., L.B., K.M., M.M.P., V.F.D., and S.F.C. contributed to the analysis and interpretation of data; and A.G.‐B., C.B., K.D., L.B., K.M., M.M.P., V.F.D., and S.F.C. contributed to the preparation of the manuscript.

## Funding

This work was supported by the Alzheimer's Association under Award Number (ARCOM‐23‐1012028 [Gilmore‐Bykovskyi]). The content is solely the responsibility of the authors and does not necessarily represent the official views of the Alzheimer's Association.

## Disclosure

The sponsor played no role in the design, methods, subject recruitment, data collection, analysis or preparation of the paper. The content is solely the responsibility of the authors and does not necessarily represent the official views of the Alzheimer's Association.

## Conflicts of Interest

The authors declare no conflicts of interest.

## Supporting information


**Text S1:** Interview guide.
**Text S2:** Follow‐up interview guide.
**Table S1:** Expanded findings by priority level, theme, and supporting quotes from participants.
**Table S2:** Original Spanish quotes and their English translations.
