# Supplementary material for: What Matters Most to People Living With Dementia and Their Care Partners During Emergency Department Visits
Source: J Am Geriatr Soc. 2025 Dec 8;74(2):396–406. doi: 10.1111/jgs.70238 (PMC12911543; doi:10.1111/jgs.70238)
Supplement: Supplementary file 1 — Text S1: Interview guide. Text S2: Follow‐up interview guide. Table S1: Expanded findings by priority level, theme, and supporting quotes from participants. Table S2: Original Spanish quotes and their English translations. [file JGS-74-396-s001.pdf]

Supplementary Text S1: Interview Guide

I. What Went Well and What Could Be Improved

1. Overall, how would you evaluate your stay at the emergency room?

2. What are some things that have gone well during your visit?

If needed, probe around environment (lights, noise), staff interactions, medical/nursing care or interventions, information, decision-making support, family involvement.

3. What are some things that could be improved about your visit?

If needed, probe around environment (lights, noise), staff interactions, medical/nursing care or interventions, information, decision-making support, family involvement.

4. I was hoping you could share a little bit about what you had hoped would happen when you came to the emergency department today. Did you have any goals in mind when you came in?

II. Priorities for Care

1. What is most important to you when you are in the emergency room?

If participant describes priorities (goals of care), probe more on their values (intangible). If they describe values, probe more on their priorities.

*For **values**, ex. "I've heard some people want to feel respected in the emergency room, is this true for you? Are there any other values you have in the emergency room?"*

*For **priorities**, ex. "I've heard some people prioritize speed and efficiency in the emergency room, is this true for you? Are there any other priorities you have in the emergency room?"*

2. What types of things do you think make good emergency room care?

3. What advice would you give people working in the emergency room when caring for someone experiencing changes in memory?

4. **PROBE FOR VIEWS ON NAVIGATOR**: Some people have mentioned the potential value of having a point person, or **navigator**, in the emergency room. What do you think about this strategy?

5. Think about someone experiencing memory changes. How might their priorities in the emergency room be different from someone without memory changes?

6. Is there anything else you would want emergency room staff to know about you or do when they care for you?

STOP: *End the interview if the participant is having trouble with questions, a clinical need/situation arises, or time is insufficient.*

III. Information and Decision-Making

1. We know there can be a lot of information shared back and forth during an emergency room visit. How do you like information to be shared with you?

2. There can also be a lot of decisions to make during an emergency room visit. How do you like to be involved or supported in decision-making?

a. *If they're with someone in the ER:* Do you like [NAME OF PERSON] or anyone else to also be involved when you're making decision?

b. *Do you feel the right people are involved in decision-making?*

c. *What are you missing by not having someone there?*

IV. Additional Priorities Questions

1. How have you shared your goals or priorities with the doctors or nurses in the emergency room?

2. Can you tell me about what makes you feel comfortable sharing your priorities with your providers in the emergency room? What kinds of things do they do or say that help you feel comfortable sharing your preferences.

3. Can you tell me about a time when you weren't comfortable sharing your preferences or priorities?

4. When you're in the emergency room, when do you think is the best time to share your priorities or preferences?

5. IF THEY HAVE A CAREGIVER: How do you prefer [name of caregiver] to be involved in sharing your preferences or priorities with the providers in the emergency room?

Supplementary Text S2: Follow-Up Interview Guide

I. Intro Script

"Thank you for your interest in a second interview!

As a reminder, our goal is to understand what matters most to people living with changes in memory and thinking and their caregivers when they are in the emergency room. This can help us develop tools to evaluate and improve the care you receive in the emergency room.

When we talked in [MONTH] while/after you were in the emergency room, you helped us understand what matters most to you. You shared [Recap, i.e. 2-3 things about what matters most to them, if they had overall positive or challenging experience].

Talking with you and others has helped us create a list of things we think matter the most for emergency room care. This list is specific to people living with changes in memory and thinking and their caregivers.

Today, I am hoping to get your feedback on this list. We are going to walk through this list piece by piece. Do you have any questions before we get started?"

Reminder to Interviewers: Assess early and often how the interview is going. If the person struggles, probe only 1-2 items in the 1st and 2nd sections and skip conditions. Probe more concrete items, and those relevant to their 1st interview. For example, instead of asking if any of the items are important, you can ask "Is a calm environment important to you?" It is OK and expected that people will reflect across multiple ER visits and/or different health encounters.

II. Things That Matter Most

Okay, so the list begins with 3 things that we heard are important for almost everyone when it comes to emergency room care. We called these "Things that Matter Most" and think they are central to people's impression or evaluation of emergency room care.
[*Read out all 3 priorities; Modulate use of first person if confusing for participant when reading out loud; Provide first laminated card if in person*]

Things That Matter Most

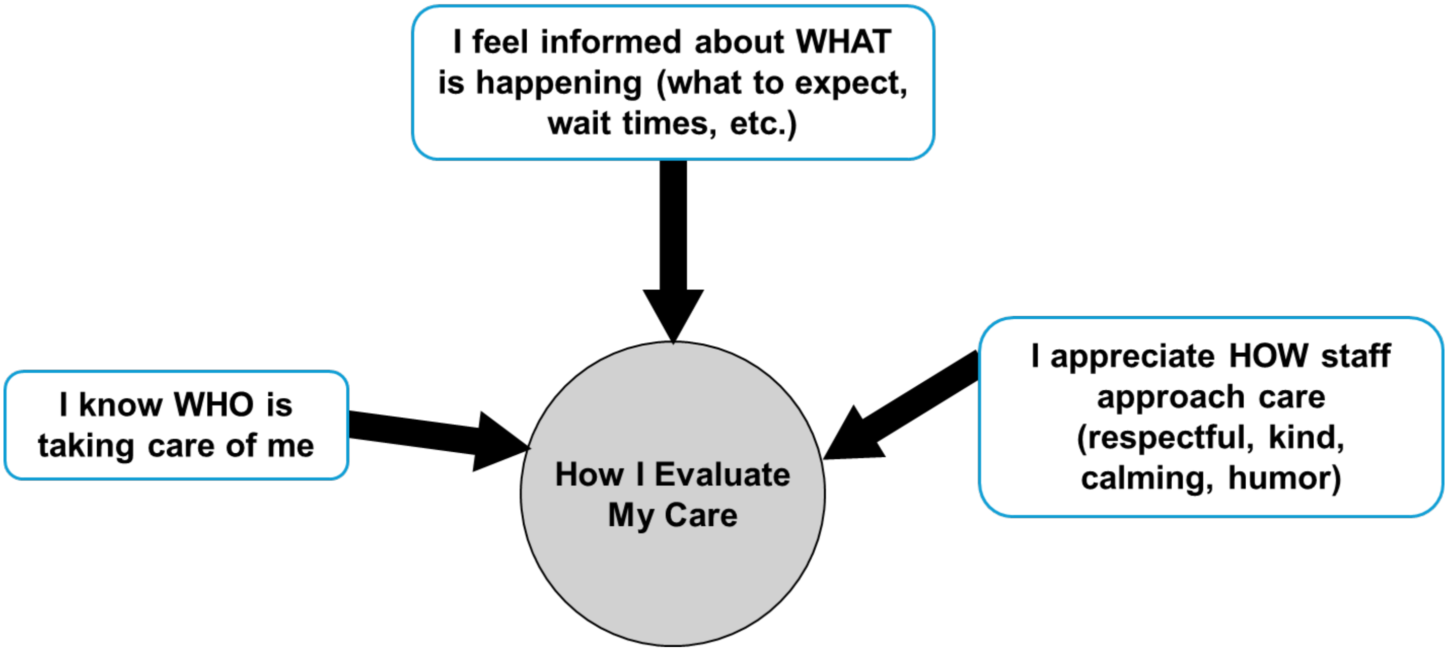

1. What do you think of this list?

2. Were any of the items on this list important to you during your emergency room visit? If yes, can you describe why?

3. Are any of these items more or less important than the others?

4. Is there anything you would change? Is there anything missing?

III. Things that Matter on a More Individual Basis

So, next, we also heard about things that mattered to some people, but not to others. We called these "Things that Matter on a More Individual Basis."
[Read out all 5 individual values; provide corresponding laminated card if in person]

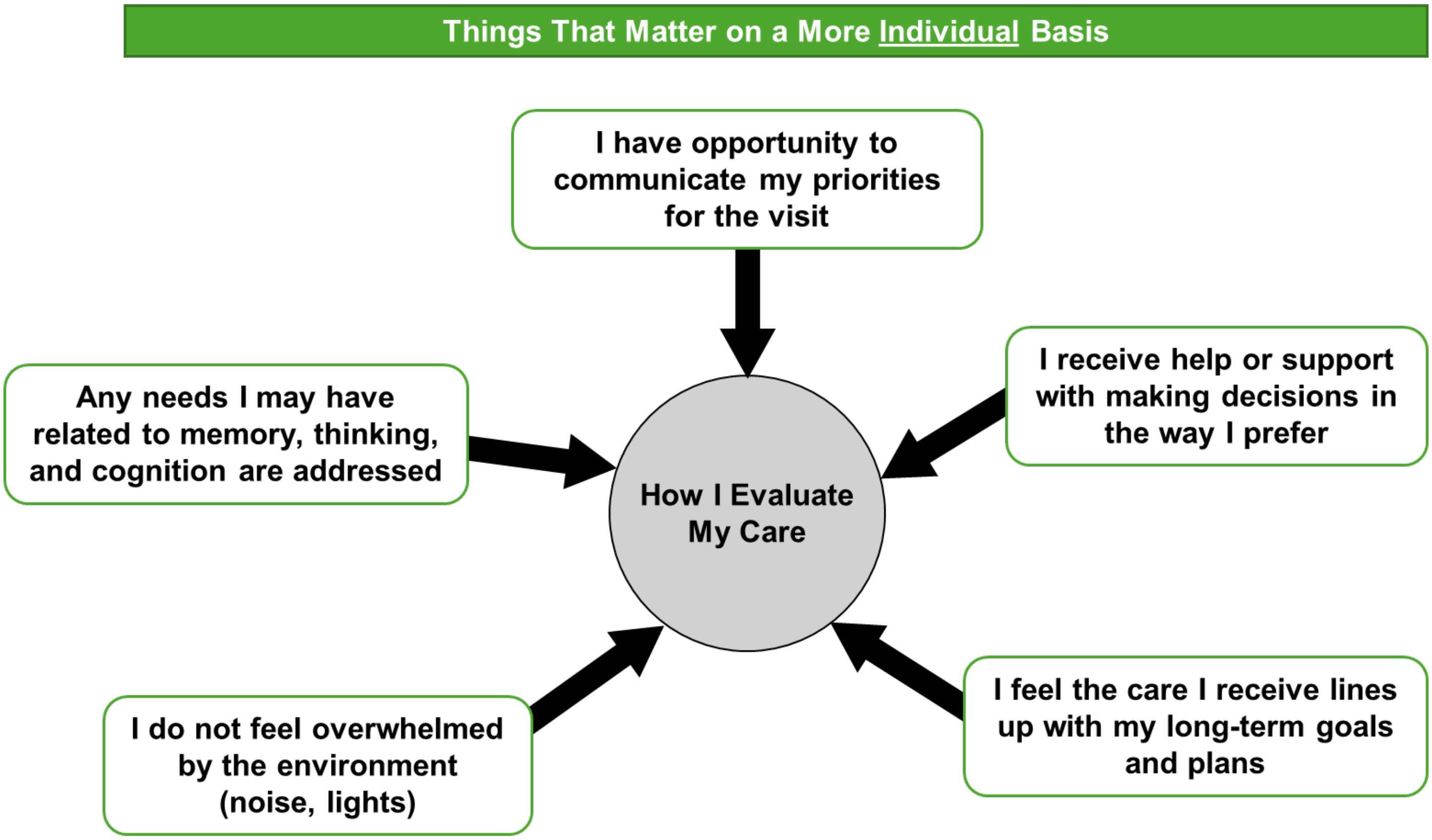

1. What do you think of this list? [Can repeat]

2. Were any of the items on this list important to you during your emergency room visit? If yes, can you describe why?

3. Are any of these items more or less important than the others?

4. Is there anything you would change? Is there anything missing?

IV. Conditions

Finally, we noticed some trends in the ways patients and caregivers would answer questions about their emergency room experience depending on their circumstances or prior experiences. For example, we noticed that patients may have different expectations for care if they have already had been to the emergency room for the same problem before. This is likely because they have knowledge about the procedures and steps are as they are occurring.

I’m going to ask you a bit more structured questions about these circumstances or conditions. It may be a bit challenging to answer some of these questions.

[Hone in on 1-2 specific conditions that are relevant to their experience]

STRUCTURED QUESTIONS

1. For all: How do you think your impression of your visit has changed over time?

2. For caregivers: Do you think your impression of the visit was different than your loved one’s? If so, how?

3. If applicable: Can you think of a time when you did not have knowledge about your condition, and how this affected your emergency room visit? What about a time you did have knowledge?

4. If applicable: Can you think of a time when you visited the emergency room for something critical? How did this affect your impression of your care? What about a time when your health condition was not as critical? How did this affect your impression of your care?

Appendix Table S1. Expanded findings by priority level, theme, and supporting quotes from participants.

Theme	Quotes
**Universal Priorities** *What matters most. Shared and uniform (generally expected of all people in the ED).*	
**Being informed at every step**<br>• Results<br>• Wait times<br>• What to expect as things unfold<br>• What's being done to me	*"They were making all kinds of decisions without explaining anything to me." (PLWD 10)*<br><br>***Interviewer:*** *When you're in the emergency room, what is most important to you?*<br>***PLWD 17:*** *Communication. Letting me know what they're doing and what's going on and what's wrong with you.*<br><br>*"And you're, you're there and you're wondering, you know, what is that like. What am I waiting for? Um. Is there good news or bad news. That sort of thing. So it's just very indefinite." (Care Partner 3)*<br><br>*"With some [PLWD], some significant percentage of their fears can be allayed to some extent if they're given a little bit of explanation of this is what we're trying to accomplish, this is how long it'll probably take roughly between that kind of thing. Explanation of what they're doing and you kind of know what they're doing, but a little bit more explanation would be helpful, I think. (PLWD 4)*<br><br>*… "if you see that we are late, let the person know...Look, we have an emergency, this person arrived who needs first aid and has priority"... When you give that to the patients… they know that [someone] is dying, well, they get priority 'cause you would wanna be priority too." (Spanish-Speaking Dyad 2 – Care Partner)*
**Knowing who is taking care of me and their role**<br>• Know who I can ask questions	*"To know who I'm seeing, their name, and what they're affiliated with. You know what type of doctor they are. And every one of them has told me you know, which is important to me. So I would like to know that." (PLWD 15)*<br><br>*"Once you get into a room, people come in and out very quickly and they don't identify themselves, they don't give their name or say if they are a nurse or a P.A. or a doctor or a resident or an attending or a specialist. Nobody identifies themselves, and it's all kind of a blur." (PLWD 10)*<br><br>*"And, um, um, one thing that I find difficult is you don't know who they are. And you don't know who's in charge. And um, so people are coming in and out and um, you know, you assume they're, most of them are emergency technicians, that they're nurses and, uh, physicians, but, uh, you're not sure who a physician is and what their role is." (Care Partner 3)*
**Care approach**<br>• Respectful<br>• Being kind & calming effect to being kind<br>• Use of humor in certain situations<br>• Feeling listened to and respected	"*The doctors, they're dedicated to their job and dedicated to their patients. You couldn't ask for a better doctors or better care than right here… They come in smiling. And I love it." (PLWD 15)*<br><br>*"This past, most recent doctor was very kind and patient and cheerful. And that was very important to her [PLWD] and her family… because it does calm a person. It calms me, it calms her … You feel respected and heard." (Dyad 1 – Care Partner)*

	“I thought the, um, staff, you know, overall were very professional and competent. And so if you don't think the people who are trying to help you are both kind and competent, it's, it's problematic. But I thought, I thought they were, you know, treated me very in a very kind and helpful fashion and were very professional and competent.” (PLWD 4)<br><br>*“They have always treated me with goodness, patience and I would say with mercy too. They have a lot of patience. They cover me, like that day when I arrived with chills, they put warm sheets on me and everything” (Spanish-Speaking Dyad 1 – PLWD)*
**Values and Preferences**<br>***More variability. Individually specific in some instances.***	
**Theme**	**Quotes**
**Goal-aligned care**<br>• Intensiveness of care (alignment/ misalignment)<br>• Efficiency<br>• Responsive to cognitive health needs<br>• Knowing what’s going on – figuring things out	*“The E.D. seems better, I guess maybe they seem a lot better than primary care doctors. They spend more time. That's just like kind of how they address problems, because I think they look to fix the problem, you know, not just say, well, I'm not feeling well, so I'll send her home and give her a few more days to see if she gets worse. So, you know, that's kind of where we're at with your primary doctor. I've seen it with my own family, too. They get older. The primary doctors don't want to do as much.” (Care Partner 2)*<br><br>*“We would have liked for them to give us a diagnosis. To this day we don't know. He had an infection, he does not know what caused it. If there is no diagnosis, it cannot be treated by the PCP” (Spanish-Speaking Dyad 2 – Care Partner)*<br><br>*“The question is, how much do you have to do? If you want to be a physician who doesn't make any mistakes, you order every test in the book. The fact that she does have a DNR bracelet on, and if she came in with a wound and nothing else, why not just treat that?” (Care Partner 3)*
**Decision-making**<br>• Supported in a way that is appropriate to my situation and preferences<br>• Being engaged in the process<br>• Right people<br>• Right timeliness<br>• Care partners in particular – feeling they are updated and engaged early on PRIOR to the PLWD being moved to the ED	*“I should be the sole one that decides about what my care is. Obviously, I can't decide the critical medical things that need to be done. I don't know. I'm not a doctor. I don't know what's going on inside my brain, inside of my body. But I just want to be included.” (PLWD 5)*<br><br>*“You'll find the need to have a family or friend or neighbor that can answer some questions that maybe the patient stalled on.” (Dyad 1 – Care Partner)*<br><br>***Care partner:*** *“Yeah. I always ask her. I mean, I can talk to the nurses, to the doctors. And to me, okay this is the best way to go. But she’s the patient. You know. Okay, these are the options. What do you think?”*<br>***PLWD:*** *“Yeah, I'm a patient but I’m also her mother. And she should have a say in it.” (Dyad 3)*
**Cognitive health-related needs taken into consideration**<br>• Triage	*“I can’t pronounce words anymore. I don’t know the meaning of them and that’s been pretty new… Show pictures to me if it’s at all possible. But like I said, if they don’t talk medical terms, then I can understand what they’re saying.” (PLWD 15)*

• Goals of care and care intensiveness<br>• Supported decision-making<br>• Supportive communication (double checking information?)<br>• Knowledge of dementia status and considerations	***Interviewer:*** *"And would there be anything you'd want people who care for you to know about just that you are experiencing memory changes?"*<br>***PLWD:*** *"They know. I try not to give them everything about me and my health. Because you know I just don't want them to worry, but they are aware that I have a memory problem and they cooperate with me on that." (PLWD 2)*<br><br>*"I don't know that they always know? She's not there for memory issues. She's there usually for gastrointestinal type things or pain. So I don't know if they put a note in the chart that this person has memory issues. If they do, then explaining, repeating themselves, I'm here to do this and then come back. I have to go and get something. When I come back, I'm here to do this. So that helps. But again, I don't know if they know to do that because I don't know if they know she has memory issues." (Care Partner 23)*<br><br>*"I think the most important thing is patience, because they explain something to you and after a while you forget it… I'm embarrassed, but I have to ask again. (Spanish-Speaking Dyad 1 – PLWD)*
**Culture and language considered**<br>• Use of interpreters<br>• Technology challenges<br>• Differences between literal interpretation and patient-centered communication<br>• Involvement of family members	*"My mom is Japanese. So sometimes I'm worried that if people keep using big words, it's hard to communicate. You got to keep things simple and to the point. She does have an accent, and it's probably harder for other people to understand her." (Care Partner 16)*<br><br>*"Yes, they have done it [interpretation] with a tablet, that is dangerous for me. I have always told them to contact me...If you don't know the person, the person's history, intimately like a family member. That you can explain to him. Knowing what the person knows, what they understand, what they like it's not the same, right?" (Spanish-Speaking Dyad 2 – Care Partner)*<br><br>*"Usually…I do the interpretation. If it's something that's technical…if it has something to do with the brain, let it be the scientific words that I don't know how to translate, then I let the interpreter translate it, but I then explain it to him [PLWD] anyway…That's what I do because sometimes, even though there are interpreters, well they have a way, the dialect is different, and you can tell." (Spanish-Speaking Dyad 2 – Care Partner)*<br><br>*"While in the ER [interpreter] was one per tablet. But since he [PLWD] didn't hear well and didn't have his hearing aids, then... I had to ask him questions, then I answered it, I listened to him or asked him the question, I simplified it or answered him then, because after that there was no longer a need for an interpreter." (Spanish-Speaking Dyad 2 – Care Partner)*<br><br>*"The thing is that because it is not in Spanish, they give me all the information in English. On the paper that they give you." (Spanish-Speaking Dyad 1 – PLWD)*<br><br>*"They explain it [treatment] to me well, because since I don't speak English, the interpreter is the one who translates for me." (Spanish-Speaking Dyad 1 – PLWD)*
**Priorities are addressed**	*"Today things have gone well. I mean she came in by ambulance so they got her in a room right away which is good, and then they started the bloodwork and urine tests and everything right away. So they got things moving pretty*

• Hydration, comfort, food needs are quickly taken care of in the ED	*quick, got the results back and things and a kind of a game plan I guess. And it's gone really smoothly today. Quickly. I've been sitting here all day which is great [laughs]." (Care Partner 2)*
**Environmental preferences**<br>• Noise<br>• Lights<br>• Specific preferences for people with memory changes	*"…your emergency room is what it is. There's all kinds of stuff happening. But just for someone with memory loss, the noise and the light are real important to keep them stable." (Care Partner 8)*<br><br>*"It's important to have everything visually marked. So the person knows where they are and where they are going. A lot of that can be done with color and numbers." (PLWD 11)*<br><br>*"You've got three very different conversations happening at the same time. For somebody who has memory problems that can be concerning. So somehow making it more, insulated, or somehow keeping the noise down. I think because when we transitioned to the neurology area, they had a nurse right outside my mom's room and it was quiet. The lights were low. I mean, it was like definitely they were focusing on that kind of a patient. And she settled right down… (Care Partner 8)*

Appendix Table S2. Original Spanish quotes and their English translations.

Original Spanish Quote in Transcript	Translated English Quote
"Ella es la que batalla conmigo, cuando estoy adolorida y eso."	"…she [PLWD's daughter] is the one who battles with me, when I am in pain." (Spanish-Speaking Dyad 1 – PLWD)
"Lo que pasa es que como no es en español, me dan toda la información en inglés."	"The thing is that because it is not in Spanish, they give me all the information in English. On the paper that they give you." (Spanish-Speaking Dyad 1 – PLWD)
… "*si tú ves que estamos atrasados…tú le dejas saber a la persona. Mira, tenemos una emergencia, llegó esta persona que necesita los primeros auxilios que tiene prioridad…Cuando tú le das eso a los pacientes…*they know that [someone] dying, well, they get priority 'cause you would wanna be priority too."	… "if you see that we are late, let the person know... "Look, we have an emergency, this person arrived who needs first aid and has priority"... When you give that to the patients… they know that [someone] is dying, well, they get priority 'cause you would wanna be priority too." (Spanish-Speaking Dyad 2 – Care Partner)
"Es que siempre me tratan con bondad, con paciencia y yo diría con misericordia también. Me tienen mucha paciencia. Me tapan, como ese día que llegue con escalofrío y me pusieron sábanas calientitas y todo eso."	"They have always treated me with goodness, patience and I would say with mercy too. They have a lot of patience. They cover me, like that day when I arrived with chills, they put warm sheets on me and everything" (Spanish-Speaking Dyad 1 – PLWD)
"Pues yo pienso que se necesita paciencia. Porque le dicen algo a uno y al rato se le olvida... Y sí, me da vergüenza, pero pues tengo que preguntar otra vez."	"I think the thing that they need is patience, because they explain something to you and after a while you forget it… And yes, I'm embarrassed, but I have to ask again. (Spanish-Speaking Dyad 1 – PLWD)
"Usually…I do the interpretation. If it's something that's technical… *si tiene que ver algo con una del cerebro, que sean las palabras científicas que yo no sé para traducir, pues entonces yo dejo que el intérprete lo traduzca, pero como quiera se lo explicó a él todavía... Eso es lo que yo hago porque a veces, aunque haya intérpretes, pues tienen una forma,* the dialect is different as you can tell."	"Usually…I do the interpretation. If it's something that's technical…if it has something to do with the brain, let it be the scientific words that I don't know how to translate, then I let the interpreter translate it, but I then explain it to him [PLWD] anyway… That's what I do because sometimes, even though there are interpreters, well they have a way, the dialect is different, and you can tell." (Spanish-Speaking Dyad 2 – Care Partner)
"Mientras en la sala de emergencias fue una por tableta Pero como él no escuchaba bien y no tenía los audífonos, entonces…había que hacer preguntas, entonces, pues yo la contestaba, yo le escuchaba o le hacía la pregunta, se la simplificaba o le contestaba entonces, pues después de eso ya no había necesidad de intérprete."	"While in the ER [interpreter] was one per tablet. But since he [PLWD] didn't hear well and didn't have his hearing aids, then... I had to ask him questions, then, well, I answered it, I listened to him or asked him the question, I simplified it or answered him then, because after that there was no longer a need for an interpreter." (Spanish-Speaking Dyad 2 – Care Partner)
"Sí lo han hecho con tableta, eso es peligroso para mí. Yo siempre le he dicho que se comuniquen conmigo…Si tú no conoces a la persona el historial de la persona, íntimamente un familiar. Que tú puedas explicarle. Mira esto es lo que ella significa, porque de asegurar lo que ellos están entendiendo lo que le estás preguntando. No es lo mismo conocer lo que lo que la persona conoce, lo que entiende, lo que lo que le gusta, no? Cómo se expresan ellos personalmente."	"Yes, they have done it [interpretation] with a tablet, that is dangerous for me. I have always told them to contact me...If you don't know the person, the person's history, intimately like a family member. That you can explain to him. Knowing what the person knows, what they understand, what they like it's not the same, right? How they express themselves personally." (Speaking Dyad 2 – Care Partner)
"Pues si me explican [treatment]. Pues ya ve, como no hablo inglés y el que me explica es el intérprete."	"They explain it [treatment] to me well, because since I don't speak English, the interpreter is the one who explains it to me." (Spanish-Speaking Dyad 1 – PLWD)
